# The Role of Single-Nucleotide Polymorphisms in Cholangiocarcinoma: A Systematic Review

**DOI:** 10.3390/cancers14235969

**Published:** 2022-12-02

**Authors:** Guanwu Wang, Lara Rosaline Heij, Dong Liu, Edgar Dahl, Sven Arke LANG, Tom Florian Ulmer, Tom LUEDDE, Ulf Peter Neumann, Jan Bednarsch

**Affiliations:** 1 Department of Surgery and Transplantation, University Hospital RWTH Aachen, 52074 Aachen, Germany; 2 Institute of Pathology, University Hospital RWTH Aachen, 52074 Aachen, Germany; 3 NUTRIM School of Nutrition and Translational Research in Metabolism, Maastricht University, 6211 LK Maastricht, The Netherlands; 4 Department of Pathology, Erasmus Medical Center Rotterdam, 3015 GD Rotterdam, The Netherlands; 5 Department of Gastroenterology, Hepatology and Infectious Diseases, University Hospital Heinrich Heine University Duesseldorf, 40225 Duesseldorf, Germany; 6 Department of Surgery, Maastricht University Medical Center (MUMC), 6229 HX Maastricht, The Netherlands

**Keywords:** cholangiocarcinoma, single-nucleotide polymorphism (SNP), cancer progression, cancer susceptibility, prognosis, systematic review

## Abstract

**Simple Summary:**

Cholangiocarcinoma (CCA) is the second most common primary liver cancer, associated with a dismal prognosis due to its late diagnosis and lack of effective systemic therapies. Single-nucleotide polymorphisms (SNPs) are polymorphisms of a DNA sequence caused by a single nucleotide variation at the genomic level between individuals. While original work investigating the role of SNPs in CCA has been published during the last decades, currently no systematic review has been conducted summarizing the current knowledge and thereby facilitating further research of this interesting topic. Thus, we here aimed to systemically evaluate and illustrate the association between SNPs and CCA, focusing on tumorigenesis and prognosis. We identified 43 SNPs in 32 genes associated with CCA risk, metastatic progression and overall prognosis based on 34 studies, and comprehensively describe the associated mechanisms and potential clinical implications within a variety of detailed figures and tables. Our findings indicate that multiple SNPs play different roles at various stages of CCA and might serve as biomarkers guiding treatment and allowing oncological risk assessment.

**Abstract:**

Single-nucleotide polymorphisms (SNPs) play an essential role in various malignancies, but their role in cholangiocarcinoma (CCA) remains to be elucidated. Therefore, the purpose of this systematic review was to evaluate the association between SNPs and CCA, focusing on tumorigenesis and prognosis. A systematic literature search was carried out using PubMed, Embase, Web of Science and the Cochrane database for the association between SNPs and CCA, including literature published between January 2000 and April 2022. This systematic review compiles 43 SNPs in 32 genes associated with CCA risk, metastatic progression and overall prognosis based on 34 studies. Susceptibility to CCA was associated with SNPs in genes related to inflammation (PTGS2/COX2, IL6, IFNG/IFN-γ, TNF/TNF-α), DNA repair (ERCC1, MTHFR, MUTYH, XRCC1, OGG1), detoxification (NAT1, NAT2 and ABCC2), enzymes (SERPINA1, GSTO1, APOBEC3A, APOBEC3B), RNA (HOTAIR) and membrane-based proteins (EGFR, GAB1, KLRK1/NKG2D). Overall oncological prognosis was also related to SNPs in eight genes (GNB3, NFE2L2/NRF2, GALNT14, EGFR, XRCC1, EZH2, GNAS, CXCR1). Our findings indicate that multiple SNPs play different roles at various stages of CCA and might serve as biomarkers guiding treatment and allowing oncological risk assessment. Considering the differences in SNP detection methods, patient ethnicity and corresponding environmental factors, more large-scale multicentric investigations are needed to fully determine the potential of SNP analysis for CCA susceptibility prediction and prognostication.

## 1. Introduction

**Figure 1:**
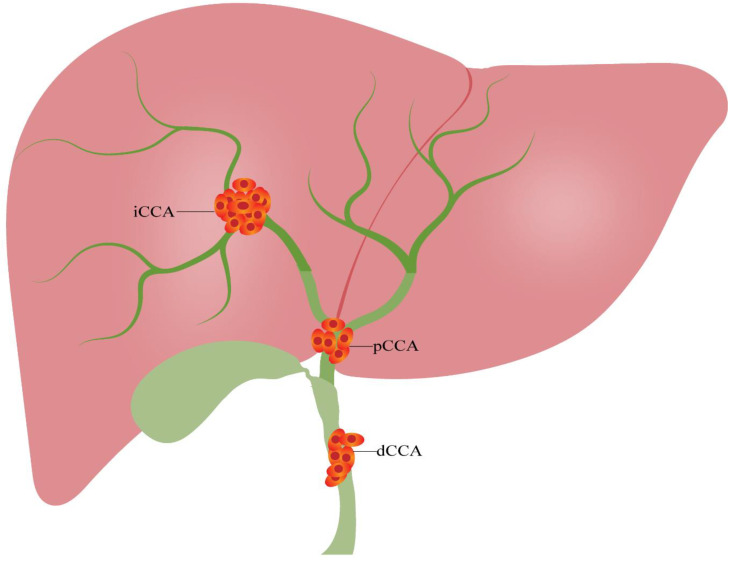
Classification of cholangiocarcinoma. Cholangiocarcinoma can be divided into three categories based on the anatomical location: iCCA, pCCA and dCCA. dCCA, distal cholangiocarcinoma; iCCA, intrahepatic cholangiocarcinoma; pCCA, perihilar cholangiocarcinoma.

Cholangiocarcinoma (CCA) is an aggressive and fatal malignancy originating from bile duct epithelial cells which is commonly associated with the requirement of complex clinical treatment and impaired oncological outcome [1,2]. Cholangiocarcinoma can be divided into intrahepatic cholangiocarcinoma (iCCA), perihilar cholangiocarcinoma (pCCA) and distal cholangiocarcinoma (dCCA) with respect to the anatomical site of origin (Figure 1) [3]. CCA is a frequently observed malignant tumor of the hepatobiliary system, accounting for about 0.7% of adult malignant tumors, 15% of all primary liver tumors and 3% of gastrointestinal cancers [4]. Interestingly, the incidence of CCA has considerably risen over the last 40 years, with the incidence in the United States tripling between 1973 and 2012 [5]. Correspondingly, in Europe, both CCA incidence and mortality increased by 9% from 1990 to 2008 [6,7]. The incidence is exceptionally high in specific regions of Asia, such as East and Southeast Asia, with more than six cases per 100,000 inhabitants per year, especially in the Mekong subregion [8]. As CCA has no noticeable early symptoms, most patients are diagnosed in advanced disease stages, which significantly limits the choice of treatment and overall prognosis [9]. Currently, surgical resection remains the only curative treatment option; however, the long-term survival and tumor recurrence rates of patients after curative-intent surgery have not improved significantly in the last decade [10,11]. Therefore, biomarkers for early detection and prognostication may have the potential to significantly improve the outcome of this disease.

**Figure 2:**
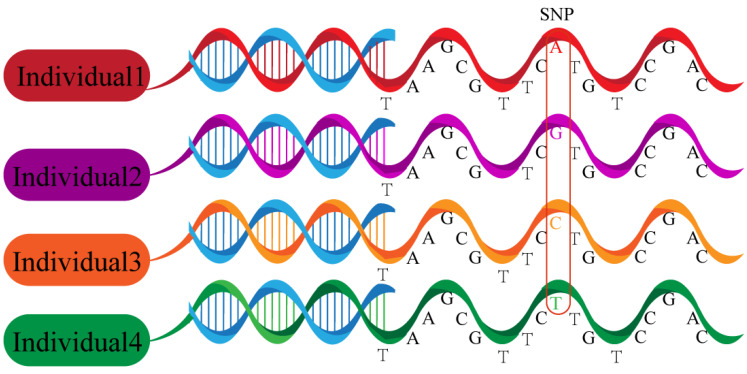
Single-nucleotide polymorphisms. Polymorphisms of a DNA sequence caused by a single nucleotide variation at the genomic level between individuals.

Single-nucleotide polymorphisms (SNPs) are polymorphisms of a DNA sequence caused by a single nucleotide variation at the genomic level between individuals (Figure 2). SNPs were discovered by Lander et al. in 1996 and became the third generation DNA genetic marker after restriction fragment length polymorphism and microsatellite polymorphism [12]. SNPs are a very common type of variation in DNA sequences, constituting more than 90% of all variation in human genomic DNA, with an average of one genotypic polymorphic SNP per thousand bases and an estimated total of up to 3 million [13]. SNPs occur in both coding and non-coding regions of a gene, with more SNPs being located in non-coding regions. SNPs in any region of a gene can potentially affect the protein structure or expression level of the gene product and thus alter an individual’s susceptibility to disease, affecting tumorigenesis and cancer progression as well as drug resistance [14]. Especially in leukemia and other hematologic diseases, a vast and growing body of literature has identified SNPs associated with cancer risk, risk of relapse, different subtypes and treatment-associated toxicity [15]. Since the completion of the sequencing of the human genome by the Human Genome Project, there has been a growing interest in studying the molecular mechanisms of CCA from a SNP perspective. As SNPs are common and have shown notable clinical relevance in other malignancies, e.g., leukemia, the aim of this review is to summarize the current status of the literature regarding the potential role of SNPs in CCA susceptibility prediction and prognostication.

## 2. Methods

### 2.1. Registration and Protocol

This review was conducted in accordance with the recent PRISMA (Preferred Reporting Items for Systematic Reviews and Meta-analyses) and registered in the International Prospective Register of Systematic Reviews (PROSPERO) with the ID CRD42022313074 [16].

### 2.2. Search Strategy

The PubMed, Embase, Web of Science and Cochrane Library databases were searched until April 2022 with the following full-text terms: “cholangiocarcinoma” OR “cholangiocellular carcinoma” OR “bile duct cancer” AND “single nucleotide polymorphism” OR “SNP” OR “polymorphism” OR “mutation” OR “allele” OR “variant” OR “variation” to identify SNPs associated with bile duct cancer risk. During the literature search, no proximity operators were used. The search was performed independently by two investigators. No additional papers were added after the citation search was completed. Unpublished literature was not considered for inclusion in this review.

### 2.3. Inclusion and Exclusion Criteria

The identified studies were screened in a multi-step approach, analyzing the title, abstract and full text. The final selection of the included studies was based on the aims of the review using the flowing criteria. The selection criteria were: (a) experiments on humans; (b) elucidation of the relationship between SNPs and cholangiocarcinoma; (c) original data; and (d) English language. Exclusion criteria were: (a) reviews, case reports, conference abstracts or letters to the editor; (b) irrelevant studies; and (c) repeated publications.

### 2.4. Data Extraction

Two independent researchers extracted the following data from included studies: the first author, publication year, country of study, patient number, sample size, study type and characteristics, gene, SNP, genotype distribution, methods of sample extraction, type of specimens, number of cases and controls and Hardy–Weinberg equilibrium results. The data were subsequently organized in standardized tables.

### 2.5. Risk of Bias in Individual Studies

All case–control studies were evaluated for quality by independent analysis of two authors. We used the Newcastle–Ottawa Scale (NOS) to assess the quality of the articles [17]. The NOS checklist contains the criteria selection (four items), comparability across groups (one item) and outcome and exposure evaluation (three items). The same three criteria were used in cross-sectional studies to assess the quality. As a result, the maximum score of the scale was nine points, with studies being categorized as low (0–3 points), moderate (4–6 points) and high quality (7–9 points), respectively. Reporting of the Hardy–Weinberg equilibrium was also considered as a quality criterion.

## 3. Results

**Figure 3:**
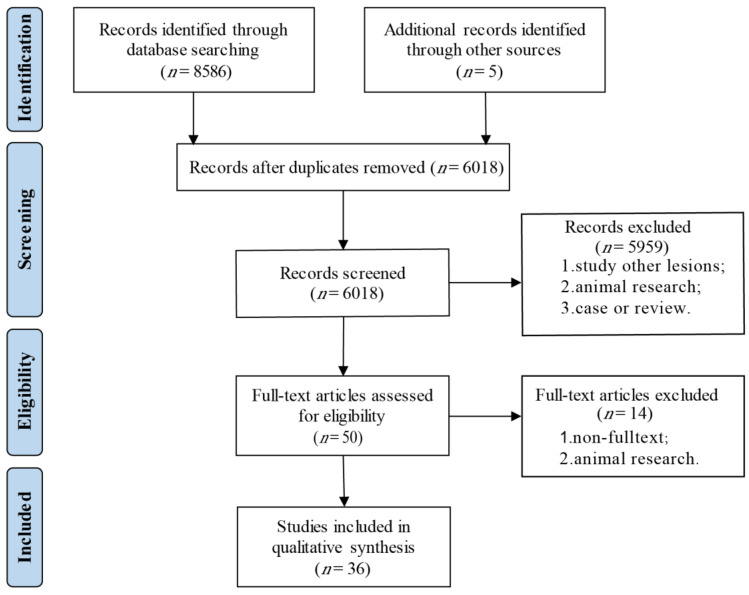
Flow diagram of the selection progress.

We retrieved 8486 articles from the PubMed, Embase, Web of Science and Cochrane library databases. After removing duplicates and abstract screening, we extracted 50 articles for a full-text assessment. After the exclusion of congress abstracts without a published full-text manuscript (*n* = 13) and one article conducting an animal experiment [18], a total of 36 papers were included in this systematic review (Figure 3). A qualitative analysis using the NOS was conducted for 24 case–control studies, displaying an overall good quality of the included studies (Table 1). Only one of the 36 publications did not report the Hardy–Weinberg equilibrium [19]. Six publications demonstrated no association with any characteristic of CCA [20,21,22,23,24,25].

### 3.1. Association of SNPs with Susceptibility to Cholangiocarcinoma

**Figure 4:**
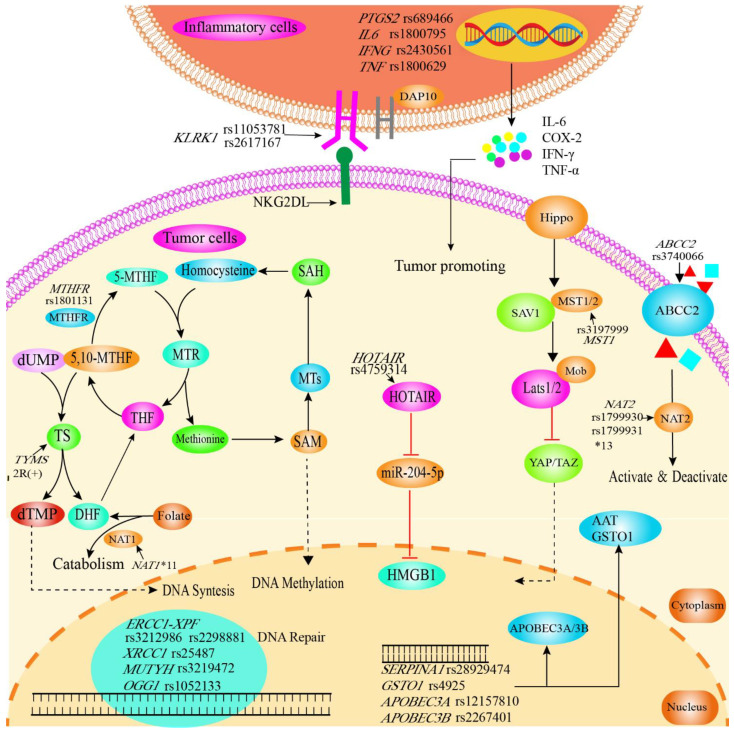
Graphical synopsis of the role of SNPs in susceptibility to cholangiocarcinoma. SNPs in genes related to inflammation, DNA repair, cellular protection against toxins, RNA, enzymes and membrane proteins are related to susceptibility. Polymorphisms in inflammatory genes (*IL6* rs1800795, *INF-γ* rs2430561, *TNF-α* rs1800629) affect levels of inflammatory cytokines, which play a key role in the inflammation-to-tumor process. The KLRK1/NKG2D receptor plays an important role in the surveillance of tumors by NK cells, and is induced by tumorigenic actions and further upregulated by chemotherapy or radiation. Polymorphisms in *KLRK1* (rs11053781, rs2617167) might therefore facilitate immune escape and tumor progression. MTHFR plays an important role in folate metabolism and is also key in maintaining the balance between DNA synthesis and methylation. *MTHFR* rs1801131 reduces the activity of MTHFR, thereby leading to folic acid deficiency and can subsequently be linked to cancer risk. The Hippo signaling pathway mainly regulates cell proliferation and apoptotic and can be affected in its activity by *MST1* rs3197999. ABCC2 is a member of the MRPs subfamily of the ABC transporter protein superfamily, transporting various molecules through extracellular and intracellular membranes. Therefore, reduced production of the ABCC2 protein by *ABCC2* rs3740066 polymorphism affects the absorption, distribution and excretion of the substrate drug or toxic substance. SNPs in *NAT1* and *NAT2* can lead to reduced expression, decreased activity and/or instability of enzymes associated with the metabolism of drugs and carcinogenic substances. HOTAIR/miR-204-5p/HMGB1 regulates cell proliferation, apoptosis and autophagy, with SNPs potentially affecting regulation. Further, SNPs in DNA repair genes can naturally be associated with cancer susceptibility (*ERCC1* rs3212986, rs2298881; *MUTYH* rs3219476; *XRCC1* rs3219472; *OGG1* rs1052133). Susceptibility to cancer is further influenced by SNPs in enzymes (*APOBEC3A* rs12157810, DNA editing; *APOBEC3B* rs2267401, RNA editing; *SERPINA1* rs28929474, abnormal expression of AAT which acts as endogenous protease inhibitor; *GSTO1* rs4925, maintenance of cellular redox homeostasis). AAT, α1-antitrypsin; ABCC2, ATP-binding cassette sub-family C member 2; APOBEC3A, Apolipoprotein B mRNA editing enzyme catalytic subunit 3A/3B; DAP10, DNAX activating protein 10; DHF, dihydrofolate acid; dTMP, deoxythymidine monophosphate; dUMP, deoxyuridine monophosphate; ERCC1, ERCC excision repair 1; GSTO1, Glutathione S-transferase omega 1; HMGB1, high mobility group box 1; HOTAIR, lncRNA HOX transcript antisense RNA; IFNG/IFN-γ, Interferon gamma; IL6/IL-6, Interleukin 6;KLRK1, Killer cell lectin-like receptor K1; LATS, large tumor suppressor kinase; miR-204-5p, microRNA-204-5p; MST1, Macrophage stimulating 1; 5, 10-MTHF, 5, 10-methylene THF; 5-MTHF, 5-methyl THF, MTHFR, methylenetetrahydrofolate reductase; MTs, methyltransferases; MTR, methionine synthase; Mob, Maps one binder; NAT1/2, N-acetyltransferase 1/2; NKG2D-L, NKG2D ligands; OGG1, 8-oxoguanine DNA glycosylase; PTGS2, Prostaglandin–endoperoxide synthase 2; SAM, S-adenosylmethionine; SAH, S-adenosylhomocysteine; SAV1, Salvador 1; TAZ, transcriptional coactivator with PDZ-binding motif; THF, tetrahydrofolate acid; SERPINA1, Serpin family A member 1; TNF-α, Tumor Necrosis Factor alpha; TS, thymidylate synthetase; XRCC1, X-ray cross complementation protein 1; YAP, Yes-associated protein.

Common risk factors for CCA include primary sclerosing cholangitis, choledochal cysts, viral hepatitis and cirrhosis, hepatolithiasis, parasitic infections and genetic polymorphisms [46]. Recent publications have drawn attention to genetic polymorphisms that may be associated with an increased epidemiological risk for CCA (Figure 4).

#### 3.1.1. Inflammation-Related Genes

Inflammatory factors, e.g., prostaglandin–endoperoxide synthase 2 (*PTGS2*, encoding COX2) produced by tumor cells or inflammatory cells, may contribute to the occurrence and development of tumors (Table 2) [47]. *PTGS2* gene polymorphisms are therefore strongly associated with gastric and nasopharyngeal cancers as well as other tumors [48]. Chaiteerakij et al. investigated 18 functional SNPs in nine genes associated with CCA risk and survival and showed that the rs2143417 allele T (Odds Ratio (OR) = 1.52, 95%-Confidence interval (95% CI) = 1.21–3.91, *p* = 0.0003) and rs689466 allele T (OR = 1.36, 95% CI = 1.10–3.69, *p* = 0.005) of *PTGS2* is significantly associated with the risk of CCA [26]. However, the results were not replicated in a subsequent case–control trial [26]. Further, *PTGS2* rs689466 has been shown to correlate with the amount of COX2 mRNA transcription [49].

Interferon gamma (IFN-γ) is a pleiotropic cytokine with anti-tumor and immunomodulatory function. In the tumor microenvironment (TME), IFN-γ plays an essential role in pro- and anti-tumor immunity. Interleukin-6 (IL-6) sustains a pro-tumor milieu by facilitating angiogenesis and tumor evasion of immune surveillance [50]. While tumor necrosis factor alpha (TNF-α) is not only extensively involved in the inflammatory response, it is also associated with tumor progression [51]. These cytokines were structurally investigated by Surapaitoon et al. in 510 patients infected with carcinogenic human liver fluke opisthorchis viverrine (OV) [41]. The patient set was further divided into individuals with advanced periductal fibrosis (APF+, *n* = 200), without advanced periductal fibrosis (APF−, *n* = 200) and diagnosed CCA (*n* = 110). Here, patients in the CCA group displayed significantly higher levels of IL-1β, IFN-γ, and TNF-α compared to the APF− and APF+ groups. *Interleukin 6* (*IL6*, encoding IL-6) rs1800795 GC genotype was significantly more abundant in the CCA group than in the AFP+ or AFP− group (APF− vs. CCA, OR= 2.35, *p* < 0.05; APF+ vs. CCA, OR = 2.95, *p* < 0.05). *Interferon gamma* (*IFNG*, encoding IFN-γ) rs2430561 AA genotype was also associated with CCA when the APF− and CCA groups were compared (OR = 2.20, *p* < 0.05) indicating that this particular genotype might cause the observed decrease in IFN-γ (Figure S1) [52]. Allele A of *tumor necrosis factor* (*TNF*, encoding TNF-α) rs1800629 was similarly associated with the risk of CCA and *IL6* rs1800795 allele C homozygous is associated with reduced IL-6 production, while the allele G homozygous is associated with increased IL-6 production (Figure S1) [53]. *TNF* rs1800629 has a significant effect on the transcriptional activity of genes. This polymorphism also influences the level of TNF gene transcriptional induction [54].

#### 3.1.2. DNA Repair Genes

DNA repair genes play a crucial role in maintaining DNA housekeeping and integrity. Polymorphisms in DNA repair genes may affect the activity of repair mechanisms and subsequently modulate the risk of developing malignancies (Table 3).

ABCC2, ATP-binding cassette sub-family C member 2; CI, confidence interval; ECCA, extrahepatic cholgangiocarcinoma; ERCC1, ERCC excision repair 1; MTHFR, Methylenetetrahydrofolate reductase; NAT1/2, N-acetyltransferases 1/2; OR, odds ratio, PCR-RFLP, Polymerase chain reaction-restriction fragment length polymorphism; PCR-HRM, Polymerase chain reaction-high-resolution melting analysis.

The *ERCC excision repair 1, endonuclease non-catalytic subunit* (*ERCC1*) gene is located on human chromosome 19q13. 2–13. 3, 15 kb in length and contains ten exons. ERCC1 is a single-stranded DNA nuclease involved in DNA strand cleavage and damage repair [55]. Sun et al. studied the effect of *ERCC1* polymorphism on the incidence of extrahepatic cholangiocarcinoma (eCCA) [40]. The authors included 127 patients with eCCA and 145 healthy individuals for analysis. The study demonstrated that the SNP rs3212986 C > A and rs2298881 A > C of *ERCC1* were associated with an increased risk of eCCA, especially in smokers.

Methylenetetrahydrofolate reductase (MTHFR) is an essential enzyme in the metabolism of folate and homocysteine, mediating the conversion of 5, 10-methylenetetrahydrofolate to 5-methyltetrahydrofolate, which provides the feedstock for intracellular DNA methylation reactions. Songserm et al. investigated the association between *MTHFR* gene polymorphisms and OV infection in the risk of developing CCA within a set of 219 patients [39]. OV-positive patients carrying the *MTHFR* rs1801131 CC variant had increased susceptibility to CCA compared to OV-negative patients carrying the wild-type *MTHFR* rs1801131 AA variant (OR = 2.0, 95% CI = 1.14–3.48). Thymidylate synthase (TS) is an enzyme encoded by the *thymidylate synthetase gene (TYMS)* that catalyzes the conversion of deoxyuridine monophosphate (dUMP) to deoxythymidine monophosphate (dTMP). A polymorphism in the dual (2R) or triple tandem repeats (3R) in the thymidylate synthase enhancer region (TSER) has been shown to affect TS expression [56]. In this context, Ko et al. showed that the combination of *MTHFR* rs1801133 CC and *TYMS* 2R(+) genotypes is more likely to increase the risk of CCA than the combination of *MTHFR* rs1801133 CC and *TYMS* 2R(−) genotypes (OR = 5.38 95% CI = 1.23–23.56 *p* = 0.0257) [31]. *MTHFR* rs1801131 can decrease the MTHFR activity and thus affects folate synthesis, with folic acid deficiency being linked to cancer risk (Figure S2) [57,58].

Base excision repair (BRE) is one of the important pathways of DNA repair systems associated with tumorigenesis [59]. Both X-ray repair cross-complementing group 1 (XRCC1) and 8-oxoguanine DNA glycosylase 1 (OGG1) are components of the BER pathway, and polymorphisms in *XRCC1* and *OGG1* are clinically important in various human cancers [60,61]. Songserm et al. investigated the effect of environmental factors and DNA repair enzyme (XRCC1 and OGG1) interactions on CCA [44]. This study included 219 CCA cases and 538 controls and showed that the combination of *XRCC1* G399A(rs25487) GG and *OGG1* C326G(rs1052133) CG was associated with susceptibility to CCA. CCA susceptibility was also increased in smokers with *XRCC1* GG compared to non-smokers with *XRCC1* GG (OR = 2.8, 95% CI = 1.30–5.91).

MutY DNA glycosylase (MUTYH) is a DNA glycosylase involved in DNA mutation repair [62]. You et al. showed that subjects with *MUTYH* rs3219472 carrying the AA genotype had a 2.8-fold higher risk of developing CCA [42]. For SNP rs3219476, individuals carrying the TG genotype showed a reduced risk of developing CCA compared to subjects with the *MUTYH* rs3219476 TT genotype (OR = 0.359, 95% CI = 0.17–0.758, *p* = 0.006).

#### 3.1.3. Cellular Protection against Toxin Genes

Regular drug use and exposure to environmental toxins may lead to cell destruction and is a commonly known risk factor in cancer [63,64]. Genes encoding enzymes such as cytochrome P450 family 1 subfamily A member 2 (CYP1A2) and N-acetyltransferases 1 and 2 (NAT1, NAT2) play a notable role in the metabolism of drugs, toxicants and endogenous compounds [65,66]. An overview of SNPs related to cellular protection against toxins in the context of CCA is provided in Table 3.

NATs catalyze the metabolism of aromatic amines through N-acetylation for detoxification. *N-acetyltransferase 1* (*NAT1*) and *N-acetyltransferase 2* (*NAT2*) gene polymorphisms can therefore lead to reduced enzyme expression, decreased activity and/or enzyme instability. Prawan et al. evaluated the association between *cytochrome P450 family 1 subfamily A member 2* (*CYP1A2*), *NAT1* and *NAT2* polymorphisms and CCA [38]. This study included 216 CCA patients and 233 healthy control subjects for genotyping, and showed that polymorphisms of NAT1*11 and *NAT2* (NAT2*6B (rs1799930), 7A (rs1799931) and *13 reduced the risk of CCA (*p* < 0.006). In addition, there was a tendency for CYP1A2*1A/*1A genotypes to reduce the risk of CCA compared with CYP1A2*1F/*1F genotypes (OR = 0.28, 95% CI = 0.08~0.94, *p* = 0.039), but this finding was only observed in male patients.

The *ATP binding cassette subfamily C member 2* (*ABCC2*) gene encodes a member of the MRPs (multidrug resistance-associated proteins) subfamily of the ABC (ATP binding cassette) transporter protein superfamily. It encodes the ABCC2/MRP2 protein, which is expressed mainly in the liver, intestine and kidney [67]. Mutations and abnormal expression of the *ABCC2* gene can lead to susceptibility to certain diseases. Hoblinger et al. investigated 60 CCA patients and 73 healthy controls [28]. Here, the frequency of *ABCC2* variant c.3972T (rs3740066) allele T was significantly higher in CCA patients (39.2%) compared to healthy controls, suggesting that *ABCC2* c.3972C > T (rs3740066) polymorphism is associated with an increased risk of CCA [28]. Brandi et al. reported an interesting example of this SNP [68]. The authors conducted genotyping of five siblings from the same family and all five were identified as *ABCC2* rs3740066 carriers. Interestingly, two out of five had a history of long-term exposure to asbestos and smoking and developed primary liver cancer (hepatocellular carcinoma or CCA), while the others showed no malignancy development. The occurrence of CCA may, in these cases, be the result of the interaction of environmental exposures and *ABCC2* rs3740066 polymorphism. *ABCC2* rs3740066 polymorphism is associated with the activity of the *ABCC2* gene promotor and affects the production of ABCC2 protein, and thus the absorption, distribution and excretion of the substrate drug or toxic substance [69].

#### 3.1.4. Other Enzyme-Related Genes

Enzymes have a wide range of roles in living organisms and are essential for various chemical reactions in cells and homeostasis, and further are also involved in the development, progression and treatment of cancer (Table 4).

*Serpin family A member 1* (*SERPINA1*) is a gene encoding α1-antitrypsin (AAT), an acute-phase protein whose abnormal expression is associated with the development and progression of various tumors. However, polymorphisms caused by the Z variant allele of *SERPINA1* results in a deficiency of the encoded protein. Mihalache et al. investigated the association of *SERPINA1* SNP rs28929474 (Z), rs17580 (S) and variant rs8004738 with CCA in 182 Caucasian patients and 350 healthy controls [37]. Here, compared to controls, SNP rs28929474 was more frequent in the CCA group (4.1% vs. 1.7%; OR = 2.46, 95% CI = 1.14–5.32; *p* = 0.036), suggesting that *SERPINA1* rs28929474 is associated with an increased risk of CCA.

*Glutathione S-transferase omega 1* (*GSTO1*) has been shown to be overexpressed in a variety of cancer cells [71,72]. Marahatta et al. investigated 30 CCA patients and 30 healthy individuals to compare their differences in *GSTO1* polymorphisms and observed a significant difference in *GSTO1* rs4925 D140 (OR = 25.13, 95% CI = 5.07–127) [73]. However, Chaiteerakij et al. failed to replicate the results of this study [26].

The *macrophage stimulating 1* (*MST1*) gene is critical in the regulation of the Hippo signaling pathway, encoding the receptor kinase RON ligand macrophage-stimulating protein (MSP, also known as MST1), and is associated with the pathogenesis of primary sclerosing cholangitis (PSC) [74]. PSC is also a significant risk factor for CCA [75]. Krawczyk et al. performed genotyping of *MST1* rs3197999 in 223 patients with CCA and 355 healthy subjects without PSC [32]. The AA genotype of the *MST1* variant rs3197999 increases the genetic risk of sporadic eCCA, independent of *MST1* gene polymorphisms leading to PSC.

The seven catalytic subunits of apolipoprotein B mRNA editing enzyme are a family of cytidine deaminases involved in innate immunity [76]. Cytidine deaminases activity causes instability and cancer in the human genome, and apolipoprotein B mRNA editing enzyme catalytic subunit 3A (APOBEC3A) is by far the most active member of this family [77]. In contrast, APOBEC3B is only weakly expressed in normal tissues but thought to be the source of somatic mutations that drive tumor development within cancer cells [78,79]. Liu et al. recruited 1240 healthy controls and 735 CCA patients for SNP analysis [45]. The results revealed that *APOBEC3B* rs2267401 genotype TG and *APOBEC3A* rs12157810 genotype CC were protective against CCA. Further, *APOBEC3A* rs1014971 was not associated with bile duct cancer compared to healthy controls but increased the risk of CCA in patients with inflammatory biliary diseases (cholangitis, cholecystitis, bile duct stone, gallstone and choledochal cyst).

#### 3.1.5. RNA-Related Polymorphisms

Both protein-coding RNA and non-coding RNA are essential for gene expression [80,81]. Polymorphisms in RNA-related genes may therefore also have an impact on tumorigenesis and prognosis (Table 4).

lncRNA *HOX transcript antisense RNA* (*HOTAIR*) is transcribed from the antisense strand of the *HOXC* gene and does not encode any functional protein. However, studies have shown that *HOTAIR* knockdowns promote the apoptosis of CCA cells cultured in vitro and reduce the migratory and invasive ability of CCA cells [82]. There are also many reports indicating that *HOTAIR* gene polymorphisms are associated with susceptibility to multiple cancers [83,84]. Lampropoulou et al. investigated the association of three *HOTAIR* SNPs (rs920778, rs4759314 and rs7958904) with CCA, including 122 CCA patients (80 men and 42 women) and 165 healthy controls [33]. *HOTAIR* SNP rs4759314 AG and GG genotypes were associated with CCA susceptibility (OR = 3.13, 95% CI = 1.65–5.91, *p* = 0.004 and OR = 12.31, 95% CI = 1.48–101.87, *p* = 0.005). In contrast, no significant association was found for SNP rs4759314 AA or rs920778 and rs7958904.

MicroRNAs (miRNAs) are non-coding RNAs involved in regulating messenger RNAs (mRNAs) after transcription and may regulate as many as up to 60% of human genes [85]. Mihalache et al. examined polymorphisms of MiR-146a in 182 CCA patients and 350 healthy individuals. Their data do not support a prominent contribution of pre-MiR-146a polymorphism in genetic susceptibility to CCA [36]. However, the MiR-146a rs2910164 GC genotype has shown a protective tendency against eCCA compared to the GG/AA genotype. However, in a meta-analysis performed by Xiong et al., MiR-146a rs2910164 polymorphism did not show a significant association with gastrointestinal cancer susceptibility [86].

#### 3.1.6. Membrane-Protein-Related Gene Polymorphisms

Studies have shown that 30% of eukaryotic-encoded proteins are membrane proteins [87]. During the transformation of normal cells into tumor cells, there may be corresponding changes in the appearance and properties of membrane proteins. Indeed, several polymorphisms in genes encoding membrane proteins have been associated with CCA (Table 4).

Activation of natural killer (NK) cells and T lymphocytes requires the involvement of killer cell lectin-like receptor K1 (KLRK1), also known as NK cell receptor G2D (NKG2D) [88]. KLRK1/NKG2D plays an important role in the surveillance of tumors by NK cells (Figure S3) [89]. Melum et al. analyzed corresponding genetic polymorphisms in 49 PSC patients with CCA [34]. Compared to controls, PSC patients with *killer cell lectin-like receptor K1* (*KLRK1*, encoding NKG2D) SNP rs11053781 and rs2617167 polymorphisms were more likely to develop CCA (OR = 2.08, 95% CI= 1.31~3.29, *p* = 0.011 and OR = 2.32, 95% CI = 1.47–3.66, *p* = 0.0020, respectively). However, Wadsworth et al. conducted a case–control study on the relationship between *KLRK1* SNPs and CCA and were not able to replicate the results [21].

Gab proteins facilitate signal transduction and translate receptor-evoked signals into different biological properties [90]. There are three known subclasses of Gab family proteins: Gab1, Gab2 and Gab3. The epidermal growth factor receptor (EGFR) is a glycoprotein with tyrosine kinase activity which regulates cell growth through autophosphorylation. If EGFR is overexpressed, it may therefore lead to uncontrolled cell growth and thus tumor formation [91]. Patients with *GRB2-associated binding protein 1* (*GAB1*) SNP rs3805246 genotype AA + GA were 2.2 times (*p* = 0.016) and patients with AA 2.0 times more likely to have CCA (*p* = 0.012). After controlling for potential confounders, patients with *EGFR* SNP rs2017000 GG + GA genotypes were 1.8 times more likely (OR = 1.772 95% CI = 1.137–3.885, *p* = 0.038), and those with GG were 1.5 times (OR = 1.530 95% CI = 1.213–2.845, *p* = 0.043) more likely, to develop CCA [35]. Another study also demonstrated the association of *EGFR* rs2017000 AA with susceptibility to CCA [70].

### 3.2. Correlation of SNPs with Cholangiocarcinoma Invasion and Metastasis

Tumor invasion and metastasis is a highly complex multi-gene regulated developmental processes involving a series of structural and functional abnormalities of related genes and functional abnormalities.

Tumor development is often accompanied by changes in cell-surface glycoproteins [92]. The polypeptide N-acetylgalactosaminyltransferases (GALNTs) family belongs to type II transmembrane proteins, which are the initiators of mucin O-glycosylation [93]. GALNT14 modifies tissue invasion and regulates migration properties [94]. Liang et al. investigated the prognostic role of *GALNT14* rs9679162 in CCA [95]. Perineural invasion and lymph node involvement were significantly higher in patients with genotype TT compared to *GALNT14* rs9679162 non-TT (*p* = 0.004 and *p* = 0.011, respectively).

Osteopontin (OPN) is a secreted viscous glycoprotein involved in chronic liver disease and is encoded by the *secreted phosphoprotein* (*SPP1*) gene [96]. OPN can increase tumor cell proliferation and reduce apoptosis, thus affecting patient survival [97]. Zhao et al. recruited 260 patients with iCCA and controlled them against a healthy cohort [43]. The authors analyzed the impact of *SPP1* −66 T/G(rs28357094), −156 G/G(rs17524488) and −443 C/T (rs11730582) polymorphisms in iCCA and found no association with the risk of CCA. However, polymorphisms in the *SPP1* rs11730582 were associated with TNM stages, metastasis and prognosis of CCA. The *SPP1* rs11730582 CT and CC genotypes were more likely to occur in TNM III + IV than in TNM I + II (*p* < 0.001). Additionally, the *SPP1* −443 CC genotype displayed an increased risk of distant metastasis compared to TT genotype (*p* < 0.01), making the *SPP1* rs11730582 polymorphism a potential predictive marker for metastastatic progression and reduced prognosis in iCCA patients.

### 3.3. Relationship between SNPs and Prognosis of Cholangiocarcinoma

**Figure 5:**
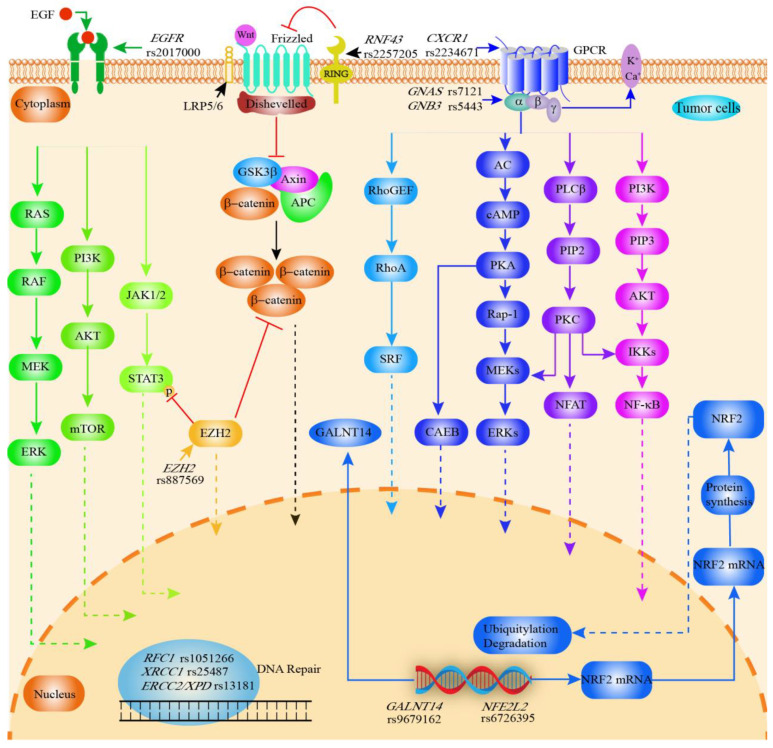
Graphical synopsis of the role of SNPs in prognosis of cholangiocarcinoma. Various SNPs are associated with clinical prognosis of CCA. *EGFR* rs2017000 affects EGFR expression and thus the EGFR signaling pathway (tumor cell proliferation, angiogenesis, tumor invasion, metastasis and inhibition of apoptosis)*. RNF43* rs2257205 inhibits the Wnt signaling pathway, while *EZH2 rs887569* inhibits the Wnt signaling and EGFR/JAK/STAT signaling pathway. G proteins are activated upon binding to ligands, initiating different signaling pathways and leading to various biological effects. *CXCR1 rs2234671 (encodes the IL8 receptor)*, *GNAS rs7121* (encodes an activated G protein alpha subunit) and *GNB3 rs5443* (encodes the β3 subunit of the G protein) are associated with G protein-coupled receptor-mediated signaling pathways. As an important transcription factor, NRF2 (encoded by *NFE2L2*) regulates antioxidant, cytoprotective, and metabolic enzymes and is involved in drug resistance as well as cancer cell proliferation. SNPs in DNA repair genes can naturally be associated with cancer prognosis (*RFC1* rs1051266; *XRCC1* rs25487; *ERCC2* rs13181). RFC1 is also associated with the replication of DNA. GALNT14 enzymes are associated with the O-glycosylation of proteins. *GALNT14* rs9617162 affects the activity of GALNT14. AC, adenylate cyclase; AKT, Protein kinase B; APC, adenomatous polyposis coli protein; cAMP, Cyclic adenosine monophosphate; CCA, cholangiocarcinoma; CXCR1, C-X-C motif chemokine receptor 1; ERK, extracellular signal-regulated kinase; EGFR, epithelial growth factor receptor; ERCC1, ERCC excision repair 1; GALNT14, polypeptide N-acetylgalactosaminyltransferase 14; GNB3, G protein subunit beta 3; GPCR, G protein-coupled receptor; GSK3β, glycogen synthase kinase 3β; IKK, I-kappa-B-kinase; JAK, The Janus kinase; mTOR, mammalian target of rapamycin; PKA, protein kinase A; MEK, Mitogen-activated protein kinase; 5-MTHF, 5-methyl THF; NFAT, Nuclear factor of activated T cells; NFE2L2, NFE2 like bZIP transcription factor 2; NF-κB, Nuclear factor kappa-light-chain-enhancer of activated B cells; NRF2, Nuclear factor erythroid 2-related factor 2; PKA, Protein kinase A; PKC, Protein kinase C; PIP2, phosphatidylinositol(4, 5)bisphosphate; PI3K, Phosphoinositide 3-kinase; PLC-β, phospholipase C beta; RAF, serine/threonine-specific protein kinases; Rap1, Ras-proximate-1; RAS, family of genes involving cellular signal transduction; RhoA, Ras homolog family member A; RNF43, Ring finger protein 43; SRF, serum response factor; STAT3, signal transducer and activator of transcription 3.

CCA treatment options are still limited, its diagnosis remains difficult, and the overall oncological prognosis is poor compared to other solid malignancies [2]. SNPs associated with prognosis are displayed in Table 5 and Figure 5.

The *G protein subunit beta 3* (*GNB3*) gene encodes the β3 subunit of the G protein. The GNB3 subunit plays a critical role in several signal transduction receptors and effectors [102]. The T allele of *GNB3* SNP 825C > T (rs5443) enhances G protein activation with increased intracellular signaling (Figure S4) [103]. Fingas et al. investigated the relationship between SNPs (*GNB3* rs5443, *BCL2*-938C > A(rs2279115), *MCL1*-386C > G) and CCA [27]. Here, patients carrying the *GNB3* rs5443 CC genotype displayed significantly longer overall survival than those with the CT or TT genotype (median survival: 31 months vs. 13 months vs. 7 months, *p* < 0.05). However, the *BCL2*-938C > A and *MCL1*-386C > G polymorphism were not associated with overall survival.

Nuclear factor erythroid 2-related factor 2 (NRF2) is a primary transcriptional regulator of genes whose products protect cells from toxicity and oxidative damage. NRF2 activation may therefore reduce cancer risk by inhibiting oxidative stress [104]. The SNPs of *NFE2 like bZIP transcription factor 2* (*NFE2L2*, encoding NRF2) rs6726395 A/G, rs2886161 C/T, rs1806649 C/T and rs10183914 C/T were analyzed by Khunluck et al. [30]. In this study, the median survival of patients with rs6726395 GG genotype (344 ± 138 (95% CI: 73–615) days) was longer compared to AA/AG genotype (172 ± 37 (95% CI: 100–244) days).

X-ray cross-complementation protein 1 (XRCC1) has a DNA repair role, mainly supporting base excision repair and single-strand break repair [105]. Pacetti et al. investigated the association of polymorphisms of *ERCC1*-C118T, *ERCC2*/*XPD*-Asp312Asn(rs1799793), *ERCC2*/*XPD* Lys751Gln(rs13181) and *XRCC1*-Arg399Gln(rs25487) with CCA [98]. In this study, the *XRCC1* rs25487 Arg/Arg genotype displayed a shorter overall survival compared to Arg/Gln and Gln/Gln genotypes (11.0 vs. 45.6 months, *p* = 0.01). For the other polymorphisms, no association was found with overall survival in this study. Additionally, Gong et al. have shown that the rs1799782 and rs25487 polymorphisms in *XRCC1* are not associated with the risk of eCCA [106].

The enhancer of zeste 2 polycomb repressive complex 2 subunit (EZH2) alters the expression of downstream target genes through trimethylation of Lys-27 in histone 3. It has been demonstrated that EZH2 is overexpressed in hypo-fractionated CCA [107]. Paolicchi et al. studied 75 patients with advanced CCA and observed a trend for lower risk of death (HR = 0.59, 95% CI 0.33–1.05, *p* = 0.075) and for longer overall survival (*p* = 0.036) in *EZH2* rs887569 TT genotype [99].

The *GNAS complex locus* (*GNAS*) gene encodes an activated G protein alpha subunit (Gsα), and polymorphisms in this gene are associated with various clinical diseases [108] (Figure S4). Schmitz et al. retrospectively genotyped 87 patients with iCCA to elucidate the potential association between *GNAS* T393C(rs7121) genotype and clinical outcomes [100]. Here, patients with iCCA carrying the *GNAS* rs7121 genotype TT displayed fewer apoptotic tumor cells and reduced OS compared to those with patients carrying the *GNAS* rs7121 genotype CT and CC (OR = 2.74, 95% CI = 1.03–7.28 *p* = 0.025).

Moruzzi et al. described the relationship between *replication factor C subunit 1* gene (*RFC1*) rs1051266 and primary liver cancers (31 hepatocellular carcinoma and 16 CCA) [109]. The results suggest that *RFC1* rs1051266 is associated with survival in primary liver cancer. The survival rate of *RFC1* rs1051266 AA was significantly lower (5-year-survival 22.2%) compared to the *RFC1* rs1051266 GG and GA genotypes (61.5% and 76%, respectively). However, this study also included a large proportion of hepatocellular, limiting the validity for the association between CCA and *RFC1* rs1051266.

*Ring finger protein 43* (RNF43) encodes a Ring family E3 ubiquitin ligase that mediates the ubiquitination of target proteins through the RING zinc finger structure and then exerts its activity through the Wnt signaling pathway [110]. RNF43 can promote the growth, proliferation and invasion of cancer cells in hepatocellular carcinoma [111]. Talabnin et al. demonstrated that RNF43 expression was reduced in iCCA and associated with rs2257205 [112]. Subsequently, overall survival was also inferior in patients with downregulated RNF43.

By binding to the receptors C-X-C motif chemokine receptor 1 (CXCR1) and C-X-C motif chemokine receptor 2 (CXCR2), IL-8 promotes cancer angiogenesis and metastasis [113]. Multiple cancer types are known to overexpress CXCR1/2 [114]. CXCR1 belongs to the GPCR family and contains seven transmembrane structural domains [115]. A study conducted by Lurje et al. examined the relationship between *CXCR1* + 860C > G(rs2234671) and overall prognosis in pCCA [101]. Here, compared to *CXCR1* rs2234671 genotype, the C allele of *CXCR1* rs2234671 has longer disease-free (*p* = 0.015), cancer-specific (*p* = 0.007) and overall survival (*p* = 0.002). In a multivariable model, the *CXCR1* rs2234671 genotype was also significantly associated with reduced DFS disease-free (RR = 3.679 95% CI = 1.399–9.672 *p* = 0.008), cancer-specific (RR = 4.957 95% CI = 1.922–12.781 *p* = 0.001) and overall survival (RR = 3.761 95% CI = 1.727–8.190 *p* = 0.001)

As mentioned above, the *GALNT14* rs9679162 TT genotype was associated with perineural infiltration and lymph node metastasis and subsequently also affecting patient survival [95]. In addition, patients with the EGFR rs2017000 AA genotype displayed significantly reduced overall survival compared to patients with GG and GG genotypes [70].

## 4. Discussion

Cholangiocarcinoma is a rare malignant tumor that is usually discovered at a late disease stage, resulting in only a minority of individuals being eligible for curative-intent surgery [1,2]. Patients with progressed disease are usually referred to palliative chemotherapy with limited clinical benefits [2]. While SNPs have been extensively investigated in various malignancies, their role in CCA remains to be defined. To the best of our knowledge, this is the first systematic review providing a comprehensive overview of the relationship between SNPs and CCA development, progression and prognostication, including a total of 44 SNPs in 33 genes. The identified genes are involved in different oncogenic pathways, including DNA repair, apoptosis or cell cycle regulation, and detoxification and display measurable effects in different stages of CCA.

Cholangiocarcinoma is not only associated with genetic but also closely related to non-genetic factors. However, SNPs can also interact with these non-genetic factors to increase the risk of CCA; for example, *GSTM1* polymorphism is not associated with CCA, but interaction with Opisthorchis viverrini (OV) increases susceptibility to CCA [22,29,116]. In smokers, *ERCC1* rs3212986 AC + CC genotype and *ERCC1* rs229888 AC + CC genotype are associated with an increased risk of extrahepatic CCA [40]. *MST1* rs3197999 homozygosity and *SERPINA1* (encoding alpha1AT) rs28929474 (Z) is more prevalent in females, and individuals carrying these SNPs are more likely to develop CCA [32,37]. In contrast, CCA risk is reduced for men carrying the CYP1A2*1A/*1A genotype [38].

Interestingly, SNPs which alone have no effect on clinical endpoints in CCA might interact together, resulting in prognostic measures. For example, polymorphisms such as *MTHFR* 677CC, *TYMS*, *XRCC1* rs25487 and *OGG1* rs1052133 do not affect susceptibility to CCA. However, *MTHFR* 677CC individuals carrying the TYMS2R(+) increase CCA risk [31]. CCA risk is also influenced by *MTHFR* gene polymorphisms in combination with raw or semi-raw freshwater fish consumption [39]. *XRCC1* rs25487 and *OGG1* rs1052133 are not associated with CCA per se, but susceptibility is increased in smokers carrying *XRCC1* GG wild-type compared to non-smokers carrying *XRCC1* GG wild-type, while CCA susceptibility was higher in GA heterozygous smokers (OR = 3.4, 95% CI = 1.60–7.28) [117]. Further, smokers carrying *OGG1* CC wild-type and CG heterozygotes are at higher risk of CCA than wild-type nonsmokers [44]. Given these numerous observations of interactions between environmental factors and SNPs, it is assumable that these genetic alterations significantly modulate the risk for cancer initiation and progression in patients which are already exposed to risk factors, e.g., smoking, alcohol or parasitic infections.

Of note, geographical background and SNPs also seem to interact in CCA. The incidence of CCA varies throughout the world, with Western countries having a significantly lower incidence than Eastern countries [118]. NKG2D is associated with tumor progression and immune recognition and is associated with nasopharyngeal carcinoma and melanoma cancer [119,120]. In the case of CCA, *KLRK1* rs11053781 and rs2617167 increased the risk of CCA in a Norwegian/Swedish PSC population, but not in a PSC cohort in the US [26,34]. In non-PSC patients, Christopher et al. showed that the *KLRK1* polymorphism was not associated with CCA in individuals from the UK. Additionally, another study based on an Asian population observed no association, underlining potential geographical differences in the significance of certain SNPs [21].

Inflammation and cancer are inextricably linked and genes regulating inflammation also influence the development of cancer [121]. Gene polymorphisms can affect cytokine levels [122]. The secretion of inflammatory factors such as IL6 (rs1800795), PTGS2 (rs2143417), TNF (rs1800629), and IFNG (rs2430561) are influenced by genetic polymorphisms, thus promoting susceptibility to CCA [26,41]. A study from Thailand confirms the association of *IL6* rs1800795 with the risk of CCA [41]; however, *IL6* rs1800797, rs2069832 and rs2069837 were not associated with the risk of CCA in a US population. According to Chaiteerakij et al., the *PTGS2* genes rs689466 and rs2143417 are associated with CCA, but their second case–control trial failed to show a statistically significant association [26]. *TNF* rs1800629 increases the risk of CCA in a Thai population, but there are no relevant studies in Western countries confirming this result in Caucasian patients. Interestingly, patients carrying genetic polymorphisms in *IL6*, *IFNG*, and *TNF* with an OV infection are also prone to CCA, underlining the above-mentioned interaction with environmental factors [41].

DNA instability is the hallmark feature of various cancers, leading to accumulation of DNA damage. DNA damage repair through nuclear excision repair (NER), base excision repair (BER), mismatch repair (MMR), homologous recombination (HR), nonhomologous end-joining (NHEJ), and translesion DNA synthesis (TLS) pathways is therefore key in maintaining DNA integrity [123]. As such, alterations in DNA damage repair capabilities play an important role in the promotion of cancer and is nowadays also suggested for targeted cancer therapy [124]. Polymorphisms in DNA repair genes are already known to affect the susceptibility to cancer in humans [39,40,125]. Our review identified that SNPs in *ERCC1* rs3212986, *MTHFR* rs1801131 and *MUTYH* rs3219476 increase the risk for the development of CCA. ERCC1 plays an important role in NER pathways by eliminating damaged DNA fragments [126]. The *ERCC1* SNP rs3212986 is also associated with lung and gastric cancer and affects mRNA levels, transcriptional stability of mRNA and ERCC1 levels [40,127,128,129]. Although it has been suggested that ERCC1 is a potential anticancer drug target, the protein has no known enzymatic activity, making the means of regulating its activity harder to decipher [130]. Thus, currently targeted therapy regarding ERCC1 has evolved for MUTYH, which is a glycosylase involved in the BER pathway and associated with colorectal cancer, and for MTHFR, interacting with folate synthesis and being prognostic in various cancers [62,131].

Systemic therapy is a cornerstone in the clinical management of CCA, as the majority of patients will not be candidates for curative-intent surgery [2]. The metabolism of chemotherapeutic drugs directly affects the effectiveness of chemotherapy. Common treatments of patients with progressed CCA are gemcitabine plus cisplatin and the combination of fluorouracil, leucovorin, and irinotecan plus oxaliplatin (FOLFIRI; FOLFIRINOX) [132]. Platinum-based drugs act as anti-tumor agents mainly by damaging the DNA of tumor cells, inhibiting replication and inducing apoptosis [133,134]. *XRCC1* rs25487 affects the activity of DNA repair enzymes and thus the sensitivity of tumor cells to platinum-based drugs [135]. In this context, overall survival was longer in Asians than Caucasians for non-small-cell lung cancer patients carrying *XRCC1* rs25487 treated with chemotherapy, including platinum-based drugs [136]. Further, increased survival after platinum-based chemotherapy regimens in breast, colorectal, non-small-cell lung, esophageal and gastric cancers carrying the *XRCC1* rs25487 allele have been observed [137,138]. Interestingly, *XRCC1* rs25487 Arg/Arg also showed a reduced survival rate in CCA, which, however, did not gain statistical significance in a small patient cohort [98]. Irinotecan can be transported out of cells via the ATP-binding cassette transporter family and ABBC2 transports the chemotherapeutic agent into the bile [139]. *ABCC2* rs3740066 is therefore associated with the metabolism of Irinotecan [140]. Hoblinger et al. demonstrated the association between *ABCC2* rs3740066 and the risk of CCA but did not analyze the prognosis of CCA [28]. In patients with CCA undergoing systemic therapy including Irinotecan, *ABCC2* rs3740066 may interact with prognosis. Activation of the NRF2 pathway facilitates resistance to radiotherapy and chemotherapy for cancer by inducing pro-survival genes, promoting cancer cell proliferation by metabolic reprogramming, repression of cancer cell apoptosis and enhancing self-renewal capacity of cancer stem cells [141,142,143]. A study from Thailand has also demonstrated that *NFE2L2* rs6726395 affects the prognosis of CCA [30]. The underlying mechanism may be related to response to therapy in these cases. *MTHFR* rs1801131 interacts with the metabolism of fluoropyrimidines and platinum drugs and is known to affect the efficiency of chemotherapy in colorectal, breast and non-small-cell lung cancers [144,145,146]. As 5-fluorouracil and cisplatin are also used in systemic therapy for CCA, this might explain the prognostic effect of *MTHFR* rs1801131 [147,148]. ERCC1 can also affect the sensitivity of cancer to chemotherapy with platinum-based drugs [149,150]. Of note, *ERCC1* rs3212986 C > A and rs2298881 A > C has already been associated with susceptibility to CCA, but unfortunately no studies have investigated the prognostic impact yet. Glycosylation is an essential modification of proteins and abnormal glycosylation is closely associated with resistance to chemotherapy [151]. Dysregulation of GALNTs expression is related to abnormal glycosylation in cancer cells, leading to chemoresistance in endometrioid, clear-cell carcinoma and HCC [152,153,154]. Interestingly, *GALNT14* rs9679162 is associated with metastasis and prognosis of CCA by a possible mechanism of *GALNT14* rs9679162-induced chemoresistance.

CCA is notoriously difficult to diagnose and differentiate from benign biliary strictures. In fact, studies of Western cohorts showed that a relevant proportion of up to 15% of patients suspected to have CCA finally showed benign histology in surgical specimens [155,156], while conventional diagnostic measures such as endoscopic retrograde cholangiopancreatography (ERCP) with brush cytology or fluoroscopy-guided biopsy display good sensitivity and specificity, and therefore negative predictive value is lacking [157]. In cases with diagnostic uncertainty, SNPs might provide an increase of accuracy in these patients. In asthma, Park et al. recently showed a sensitivity of 64.7% and specificity of 85.0%, with 42.1% positive and 93.4% negative predictive values for a summed risk score of 14 SNPs for diagnosis [158]. It is therefore assumable that a combination of clinical characteristics and SNPs might also be useful in CCA, but this approach warrants further research in the near future.

Our systematic review has certain limitations based on the available set of literature. First, the majority of articles did not distinguish between iCCA and pCCA, which are nowadays considered to be biologically distinct cancers with different genetic backgrounds and should therefore be addressed separately in future studies [159]. Second, most articles did not comprise subgroup analysis of the interaction of SNPs with age, gender, environmental factors and other SNPs. Third, the number of studies focusing on prognosis in case of CCA with the potential to guide treatment selection and patient management is unfortunately limited and warrants further research.

## 5. Conclusions

Considering these limitations, we comprehensively summarize multiple SNPs increasing susceptibility to CCA and interacting with clinical outcome in different stages of CCA. SNPs have the potential to identify patients prone to developing CCA, in particular in combination with other clinical characteristics or environmental factors. Further, in patients with CCA, SNPs might be used for prognostic purposes. Given the differences in SNP detection methods and patient ethnicity and environment, further studies with large samples are needed to assess all variables associated with CCA. Future studies are necessary to unravel the full potential of SNPs in the context of CCA. A major focus should be on the improvement of early diagnosis of CCA, especially in cases with indeterminate biliary strictures or high-risk patients, e.g., primary sclerosing cholangitis. Here, the combination of clinical features and SNP profiles have the potential to improve diagnostic accuracy. Given the plethora of future systemic therapies for CCA with traditional chemotherapy being accompanied by targeted antibodies or immunotherapy, SNPs associated with drug and receptor metabolism should also be researched in order to tailor therapy for these complex patients.

## Figures and Tables

**Table 1:** Quality assessment of included studies. Newcastle–Ottawa scale quality analysis of 23 case–control studies on the relationship between SNP and cholangiocarcinoma [17].The maximum score of the scale is nine points, with studies being categorized as low (0–3 points), moderate (4–6 points) and high quality (7–9 points), respectively. (★) represents one point.

Author/Year/Ref.	Selection	Comparability	Outcomes	Quality Score
Chaiteerakij, 2015 [26]	★★★★	★★	★★★	9/9
Fingas, 2010 [27]	★★★★	★★	★★★	9/9
Hoblinger, 2009 [28]	★★★★	★	★★★	8/9
Honjo, 2005 [29]	★★★★	★	★★★	8/9
Khunluck, 2014 [30]	★★★★	★★	★★★	9/9
Ko, 2006 [31]	★★★★	★★	★★★	9/9
Krawczyk, 2013 [32]	★★★★	★★	★★★	9/9
Krawczyk, 2011 [19]	★★★	★	★★★	7/9
Lampropoulou, 2021 [33]	★★★★	★★	★★★	9/9
Melum, 2007 [34]	★★★★	★	★★★	8/9
Meng, 2014 [35]	★★★★	★	★★	7/9
Mihalache, 2012 [36]	★★★★	★★	★★★	9/9
Mihalache, 2011 [37]	★★★★	★★	★★★	9/9
Prawan, 2005 [38]	★★★★	★	★★★	8/9
Songserm, 2011 [39]	★★★★	★★	★★★	9/9
Sun, 2018 [40]	★★★★	★	★★★	8/9
Surapaitoon, 2017 [41]	★★★★	★★	★★★	9/9
Wadsworth, 2019 [21]	★★★★	★★	★★★	9/9
You, 2012 [42]	★★★★	★★	★★★	9/9
Zhao, 2014 [43]	★★★★	★★	★★★	9/9
Zeng, 2013 [22]	★★★★	★★	★★★★	9/9
Songserm, 2014 [44]	★★★★	★★	★★★★	9/9
Liu et al., 2022 [45]	★★★★	★★	★★★★	9/9
Hsing et al., 2008 [25]	★★★★	★★	★★★	8/9

**Table 2:** The relationship between the SNPs in inflammation-related genes with the susceptibility of CCA. Various studies investigated SNPs in inflammation-related genes related to the susceptibility of CCA.

Gene	SNP	Factor	Method	Sample	Case (%)	Control (%)	OR (95% CI)	*p* Value	Susceptibility	Reference
*PTGS2*	rs689466	C	TaqMan	Blood	163 (22)	252 (17)	1.36 (1.10–1.69)	0.005	Increase	Chaiteerakij et al., 2015 [26]
	rs2143417	T	TaqMan	Blood	148 (20)	207 (14)	1.52 (1.21–1.91)	0.0003	Increase	
*IL6*	rs1800795	GC	PCR-RFLP	Blood	45 (40.9)	67 (33.5)	2.35 (1.31–4.21)		Increase	Surapaitoon et al., 2017 [41]
		GC	PCR-RFLP	Blood	45 (40.9)	59 (29.5)	2.95 (1.64–5.31)		Increase	
		C	PCR-RFLP	Blood	111 (50.5)	93 (23.3)	3.36 (2.32–4.85)		Increase	
*IFNG*	rs2430561	AA	PCR-RFLP	Blood	54 (49.1)	65 (32.5)	2.20 (1.13–4.20)		Increase	
*TNF*	rs1800629	A	PCR-RFLP	Blood	173 (78.6)	284 (71.0)	1.50 (1.00–2.26)		Increase	
		A	PCR-RFLP	Blood	173 (78.6)	278 (69.5)	1.61 (1.08–2.43)		Increase	

CI, confidence interval; PTGS2, Cyclooxygenase-2; IL6, Interleukin 6; IFNG, Interferon gamma; TNF, Tumor necrosis factor; OR, odds ratio; PCR-RFLP, Polymerase chain reaction–restriction fragment length polymorphism.

**Table 3:** The relationship between SNPs in DNA repair genes and cellular protection against toxin genes with the susceptibility of CCA. Various studies investigated the SNPs of DNA repair genes and cellular protection against toxin genes related to the susceptibility of CCA. NAT2*6B, 590G > A (rs1799930); NAT2*7A, 857G > A (rs1799931); NAT1*11 and NAT2*13.

Gene	SNP	Factor	Method	Sample	Case (%)	Control (%)	OR (95% CI)	*p* Value	Susceptibility	Reference
*ERCC1*	rs3212986(ECCA)	AC + AA	PCR-RFLP	Blood	68 (53.5)	59 (40.7)	1.68 (1.04–2.72)	0.03	Increase	Sun et al., 2018 [40]
*MTHFR*	rs1801131	CC	PCR-HRM	Tissues	62 (35.4)	72 (20.6)	2.00 (1.14–3.48)		Increase	Songserm et al., 2011 [39]
*NAT1*		* 11	PCR-RFLP	Blood	1 (0.2)	11 (2.4)	0.10 (0.00–0.58)	0.005	Decrease	Prawan et al., 2005 [38]
*NAT2*		* 13	PCR-RFLP	Blood	8 (1.9)	24 (5.2)	0.35 (0.16–0.77)	0.008	Decrease	
	rs1799930	* 6B	PCR-RFLP	Blood	6 (1.4)	22 (4.7)	0.28 (0.12–0.69)	0.004	Decrease	
	rs1799931	* 7A	PCR-RFLP	Blood	9 (2.1)	28 (6.0)	0.33 (0.16–0.70)	0.003	Decrease	
*MUTYH*	rs3219476	TG	PCR-RFLP	Blood	20 (30.9)	58 (58.0)	0.36 (0.17–0.76)	0.006	Decrease	You et al., 2013 [42]
	rs3219472	AA	PCR-RFLP	Blood	12 (20.3)	7 (7.0)	2.82 (0.99–8.00)	0.047	Increase	
*ABCC2*	rs3740066	T	TaqMan	Blood	47 (39.2)	38 (26.0)	1.83 (1.09–3.08)	0.022	Increase	Höblinger et al., 2009 [28]

**Table 4:** SNPs in other genes associated with susceptibility to CCA. Various studies investigated the gene SNPs related to the susceptibility of CCA.

Gene	SNP	Factor	Method	Sample	Case (%)	Control (%)	OR (95% CI)	*p* Value	Susceptibility	Reference
*SERPINA1*	rs28929474 (Z)	T	TaqMan	Blood	15 (4.0)	12 (2.0)	2.46 (1.14–5.32)	0.036	Increase	Mihalache et al., 2015 [37]
*GSTO1*	rs4925	D140	PCR	Tissues	18 (30.0)	4 (6.7)	8.50 (2.07–37.85)	<0.05	Increase	Marahatta et al., 2005
*MST1*	rs3197999	GG	TaqMan	Blood	115 (52.0)	194 (55.0)	1.97 (1.09–3.36)	0.023	Increase	Krawczyk et al., 2013 [32]
		AA (ECCA)	TaqMan	Blood	22 (12.0)	24 (6.0)	2.04 (1.09–3.84)	0.024	Increase	
*HOTAIR*	rs4759314	GG	PCR-RFLP	Blood	7 (5.7)	1 (0.6)	12.31 (1.48–101.87)	0.005	Increase	Lampropoulou et al., 2021 [33]
		AG	PCR-RFLP	Blood	32 (26.3)	18 (10.9)	3.13 (1.65–5.91)	0.0004	Increase	
*KLRK1*	rs11053781	G/A	TaqMan	Blood	32 (66.0)	184 (50.0)	1.95 (1.23–3.07)	0.0038	Increase	Melum et al., 2007 [34]
	rs2617167	A/G	TaqMan	Blood	19 (39.0)	85 (23.0)	2.20 (1.40–3.44)	0.00046	Increase	
*GAB1*	rs3805246	AA + AG VS GG	TaqMan	Tissues	154 (68.4)	71 (31.6)	2.15 (1.28–3.71)	0.016	Increase	Lingqin et al., 2014 [35]
		AA VS (AG + GG)	TaqMan	Tissues	35 (15.5)	190 (84.5)	1.98 (1.21–2.84)	0.012	Increase	
*EGFR*	rs2017000	AA	TaqMan	Blood	26 (11.6)		1.92 (1.14–2.59)	0.002	Increase	Lingqin et al., 2015 [70]
*APOBEC3B*	rs2267401	TG	Taqman	Blood	***	***	0.51 (0.36–0.72)	0.00016	Decrease	Liu et al., 2022 [45]
		TG + GG	Taqman	Blood	***	***	0.69 (0.51–0.94)	0.0189	Decrease	
*APOBEC3A*	rs12157810	CC	Taqman	Blood	***	***	0.44 (0.33–0.60)	<0.001	Decrease	
		AC + CC	Taqman	Blood	***	***	0.80 (0.66–0.97)	0.025	Decrease	

*** No data shown in the original publication. APOBEC3, Apolipoprotein B mRNA editing enzyme-catalytic polypeptide-like 3; EGFR, Epidermal growth factor receptor; GAB1, GRB2 associated binding protein 1; GSTO1, Glutathione S-transferase omega 1; HOTAIR, lncRNA HOX transcript antisense RNA; MST1, Macrophage stimulating 1; KLRK1, Killer cell lectin-like receptor K1; OR, Odds ratio; SERPINA1, Serpin family A member 1.

**Table 5:** Relationship between SNPs and clinical prognosis of cholangiocarcinoma. Various studies investigated the association between SNPs and oncological prognosis of cholangiocarcinoma.

Gene	SNP	Factor	Method	Samples	Case (%)	Control (%)	*p* Value	Prognosis (OS)	Reference
*GNB3*	rs5443	CC VS (CT + TT)	PCR	Blood	17 (42.5)	23 (57.5)	<0.05	Improved	Fingas et al., 2009 [27]
*NFE2L2*	rs6726395	GG VS (AA/GG)	TaqMan	Blood	34 (38.6)	54 (61.4)	0.006	Improved	Khunluck et al., 2014 [30]
*GALNT14*	rs9679162	TT VS (TG + GG)	Sanger sequencing	Tissues	35 (31.3)	77 (38.7)	0.023	Reduced	Liang et al., 2017 [95]
*EGFR*	rs2017000	AA VS (GG + GA)	TaqMan	Blood	21 (10.2)	105 (88.8)	0.021	Reduced	Lingqin et al., 2015 [70]
*XRCC1*	rs25487	Arg/Arg VS(Arg/Gln + Gln/Gln)	TaqMan	Blood	17 (51.5)	16 (48.5)	0.013	Reduced	Pacetti et al., [98]
*EZH2*	rs887569	TT VS (CC + CT)	TaqMan	Blood	***	***	0.036	Improved	Paolicchi et al., 2013 [99]
*GNAS1*	rs7121	TT VS (CT + CC)	PCR	Tissues	7 (14.0)	40 (85.1)	<0.008	Reduced	Schmitz et al., 2007 [100]
*CXCR1*	rs2234671	CC VS CG	PCR-RFLP	Tissues	92 (83.6)	18 (16.4)	0.002	Improved	Lurje et al. [101]

*** No data shown in the original. CXCR1, C-X-C motif chemokine receptor 1; EGFR, Epidermal growth factor receptor; EZH2, Enhancer of zeste 2 polycomb repressive complex 2 subunit; GALNT14, Polypeptid N-acetylgalactosaminyltransferase 14; GNB3, G protein subunit beta 3; NFE2L2, NFE2 like bZIP transcription factor 2; OS, overall survival; RFC1, Replication factor C subunit 1; XRCC1, X-ray cross complementation protein 1.

## Supplementary Materials

The following supporting information can be downloaded at: https://www.mdpi.com/article/10.3390/cancers14235969/s1, Figure S1: Signaling pathways of *IL6* and *IFNG* and SNPs influencing signaling by affecting the secretion of IL-6 and IFN-γ; Figure S2: Mechanism of *MTHFR* A1298C (rs1801131) affecting DNA synthesis and methylation; Figure S3: Function of *KLRK1* SNP in NK and T cells; Figure S4: Mechanism of action of *GNAS1* T393C and *GNB3* rs5443 in the G-protein coupled receptor (GPCR) signaling pathway.
