# Supplementary material for: The Role of Single-Nucleotide Polymorphisms in Cholangiocarcinoma: A Systematic Review
Source: Cancers (Basel). 2022 Dec 2;14(23):5969. doi: 10.3390/cancers14235969 (PMC9739277; doi:10.3390/cancers14235969)
Supplement: Supplementary file 1 [file cancers-14-05969-s001.zip › cancers-2014511-supplementary.pdf]

Supplementary Materials

The Role of Single-Nucleotide Polymorphisms in Cholangiocarcinoma: A Systematic Review

Guanwu Wang, Lara Rosaline Heij, Dong Liu, Edgar Dahl, Sven Arke LANG, Tom Florian Ulmer, Tom LUEDDE, Ulf Peter Neumann and Jan Bednarsch

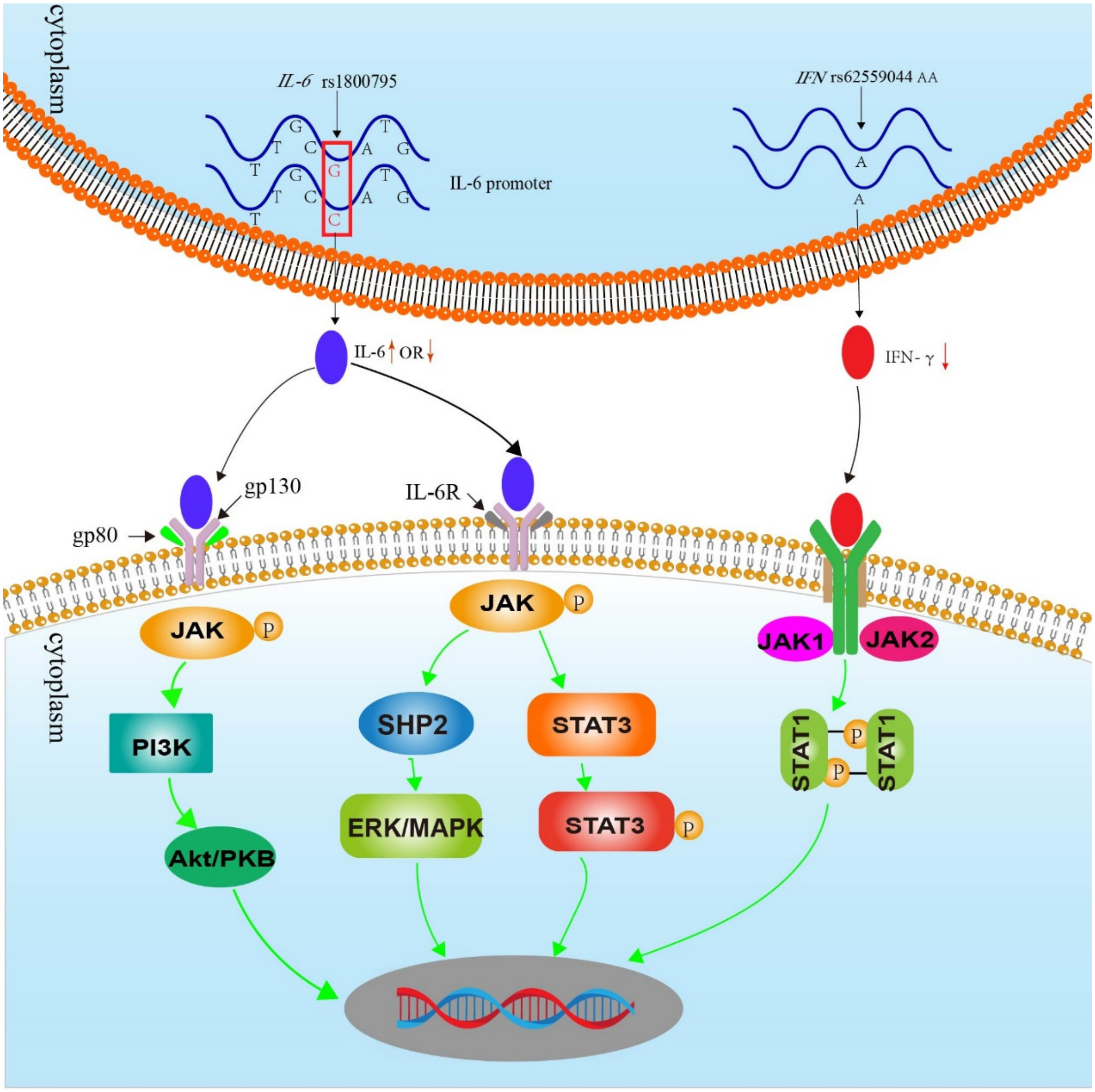

Figure S1. Signaling pathways of IL6 and IFNG and SNPs influencing signaling by affecting the secretion of IL-6 and IFN-γ. The *IL6* gene is located at chromosome 7p21-24 with the *IL6* rs1800795 polymorphism being located upstream of the *IL6* gene promoter. The polymorphism *IL6* rs1800795 does affect plasma levels of IL-6 which interacts with the gp130 and gp80 receptors. The downstream pathways PI3K- PKB/Akt, JAK/STAT3 and Ras/MAPK can subsequently cause cell proliferation, tumor angiogenesis and metastasis. IFNG is also closely related to tumor proliferation and tumor immune escape. *IFNG* rs2430561 AA genotype results in reduced levels of IFN-γ in plasma. The most important pathway for IFN-γ to generate signaling is the JAK/ STAT pathway. ERK, Extracellular regulated protein kinases; IFNG/IFN-γ, Interferon gamma; IL6/ IL-6, Interleukin 6; IL-6R,IL-6Rα (gp80 or CD126); IL-6Rβ (gp130 or CD130); JAK, Janus kinase; MAPK, Mitogen-activated protein kinase; STAT1/3, Signal transducer and activator of transcription 1/3; PI3K, Phosphoinositol-3 kinase; PKB, Protein kinase B.

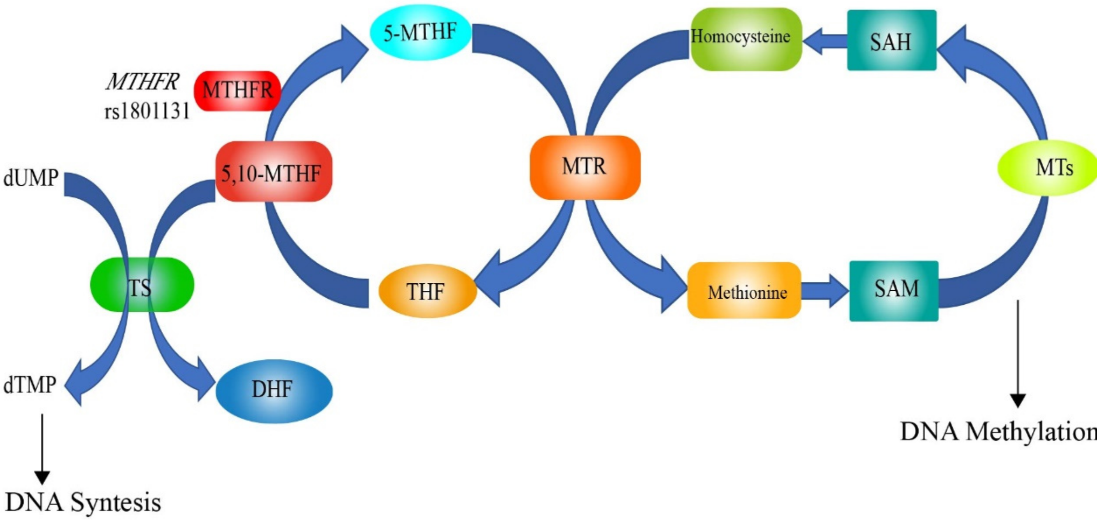

Figure S2. Mechanism of *MTHFR* A1298C (rs1801131) affecting DNA synthesis and methylation. MTHFR plays an important role in folate metabolism and is also key in maintaining the balance between DNA synthesis and methylation. MTHFR catalyzes the reduction 5,10-MTHF to 5-MTHF. *MTHFR* rs1801131 reduces the activity of MTHFR thereby affecting the production of 5-MTHF and leading to substrate accumulation and reduced folate levels. Folic acid deficiency can subsequently be linked to cancer risk. dUMP, deoxyuridine monophosphate; dTMP, deoxythymidine monophosphate; DHF, dihydrofolate acid; Hcy, homocysteine; MTHFR, methylenetetrahydrofolate reductase; MTs, methyltransferases; MTR, methionine synthase; SAM, S-adenosylmethionine; SAH, S-adenosylhomocysteine; THF, tetrahydrofolate acid; 5-MTHF,5,10-methylene THF; 5-MTHF, 5-methyl THF.

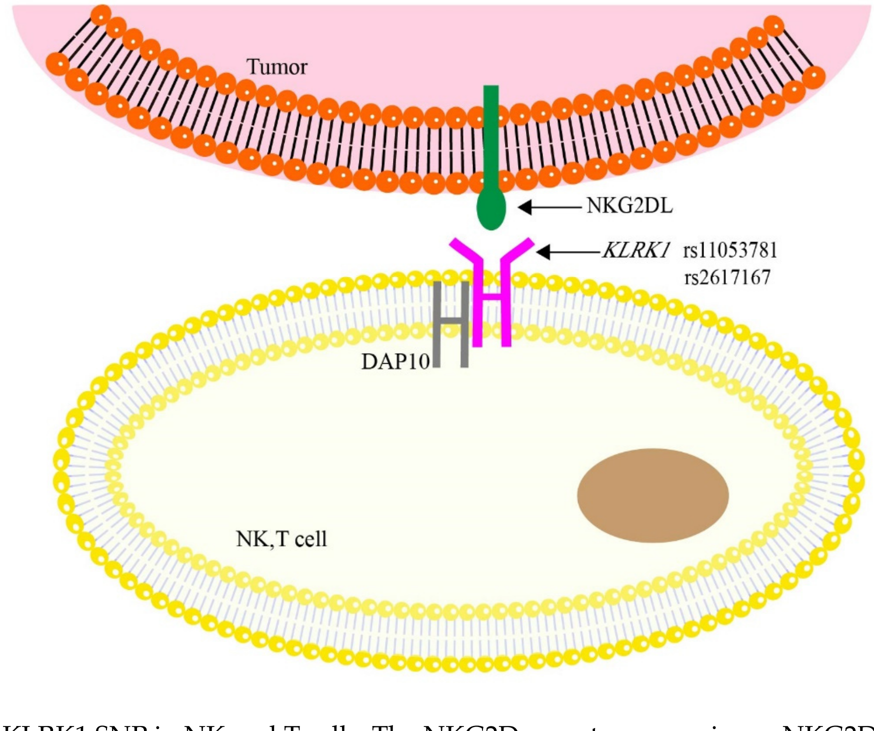

Figure S3. Function of KLRK1 SNP in NK and T cells. The NKG2D receptor recognizes a NKG2D ligands which are generally absent on the surface of normal cells but are induced by tumorigenic actions and might also further upregulated by chemotherapy or radiation. NKG2D ligands on tumor cells can activate NKG2D receptors NK cells and T

cells resulting in cell-mediated destruction. Polymorphisms in *KLRK1* (rs11053781, rs2617167) might therefore facilitate immune escape and tumor progression. DAP10, DNAX activating protein 10; KLRK1, Killer cell lectin like receptor K1; NKG2D, the natural killer group 2, member D;NKG2D-L,NKG2D ligands.

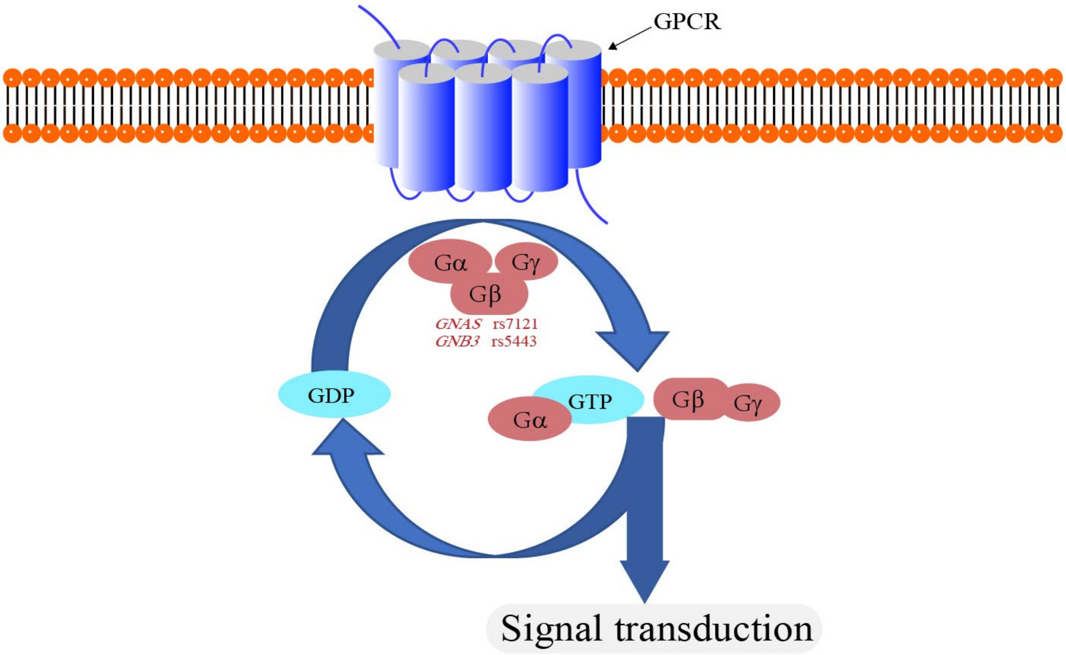

Figure S4. Mechanism of action of *GNAS1* T393C and *GNB3* rs5443 in the G-protein coupled receptor (GPCR) signaling pathway. The *GNB3* gene encodes the β3 subunit of the G protein. The *GNAS* gene encodes an activated G protein alpha subunit (Gsα). The T allele of the *GNAS* rs7121 polymorphism can cause increased expression of Gαs mRNA in a variety of tissues. *GNB3* rs5443 allele T increases G protein-mediated signal transduction by affecting Gβ activity. G protein-coupled receptors (GPCRs), in response to agonists, can transmit extracellular signals to the intracellular compartment, triggering a range of physiological activities. The *GNAS* rs7121 and *GNB3* rs5443 polymorphism can cause abnormal physiological activities, such as tumorigenesis. GPCR, G-protein coupled receptor; GDP, Guanosine-5'-diphosphate; GTP, Guanosine triphosphate; GNB3, G protein subunit beta 3, GNAS,GNAS complex locus.
